# Exploring Knowledge and Awareness of HCV Infection and Screening Test: A Cross-Sectional Survey Among an Italian Sample

**DOI:** 10.1007/s10900-023-01218-4

**Published:** 2023-04-28

**Authors:** Giuseppina Lo Moro, Giacomo Scaioli, Lorenzo Vola, Laura Guastavigna, Roberta Frattin, Elisabetta De Vito, Fabrizio Bert, Roberta Siliquini

**Affiliations:** 1 Department of Public Health Sciences and Pediatrics, University of Turin, Turin, Italy; 2 Health Local Unit “ASL TO3”, Turin, Italy; 3 Department of Human, Social and Health Sciences, University of Cassino and Southern Lazio, Cassino, Italy; 4 A.O.U. City of Health and Science of Turin, Turin, Italy

**Keywords:** HCV, HCV screening, Knowledge, General population

## Abstract

Addressing HCV represents a public health priority, especially in Italy, which has the highest HCV prevalence in Europe. This study primarily aimed to explore knowledge about the HCV infection and awareness of the existence of the HCV screening test in Italy, before the implementation of awareness campaigns in 2022. An online cross-sectional survey was conducted (December 2021–January 2022). The primary outcomes were: Disease Knowledge Score (DKS), Prevention and Transmission Knowledge Score (PTKS) (for both: scoring from 0 to 100%, higher scores corresponded to higher knowledge), and being unaware of the existence of the HCV screening. The final sample consisted of 813 participants. The median DKS was 75% (IQR = 66.7–83.3), the median PTKS was 46.2% (IQR = 38.5–53.8), and 23.2% of participants were unaware of HCV screening. Higher education, health-related study or profession, history of accidental injuries, being affected by HCV and having actively searched for information on HCV had positive associations with DKS. LGBT males showed significantly lower DKS. Considering PTKS, participants affected by HCV the disease had a negative association with this score. Having a postgraduate education reduced the likelihood of not knowing about the HCV screening test, while having at least one family member affected by hepatitis C increased this probability. This study highlighted a concerning lack of knowledge about prevention and transmission, indicating a need for targeted education campaigns. The findings emphasized the importance of information and motivation and identified male LGBT + individuals as a vulnerable group with limited disease knowledge. Future research should concern the effectiveness of awareness campaigns.

**Supplementary Information:**

The online version contains supplementary material available at 10.1007/s10900-023-01218-4.

## Introduction

Hepatitis C represents an important global public health priority, as it is responsible for 71.1 million chronic infections, 1.75 million new infections per year and over 340,000 deaths per year [1–3]. In the European Union (EU), an estimated 3.9 million people have chronic hepatitis C virus (HCV) infection [4], which is seven times more prevalent than human immunodeficiency virus (HIV) infection [5]. Additionally, 300,000 new cases and 64,000 deaths from HCV infection are recorded each year [6]. Furthermore, several studies have shown that the morbidity and mortality rates from hepatitis C may be underestimated, as the infection often progresses asymptomatically or it is responsible for extra-hepatic complications that makes the diagnosis of HCV infection more difficult [7–9].

Without a greater investment in diagnostic testing and treatment, the virus is expected to continue to spread globally reaching 13 million new cases and 1.1 million deaths by 2030 [2]. For this reason, and thanks to the introduction in 2011 of Directly Acting Antivirals (DAAs), which have revolutionized therapeutic perspectives allowing healing in over 95% of cases treated, in May 2016 the World Health Organization (WHO) approved the Global Health Sector Strategies (GHSS—2016–2021) that aim to achieve elimination of viral hepatitis by 2030 [10]. Consequently, many countries adopted these recommendations and launched campaigns to eliminate the infection.

Italy, which has the highest prevalence of HCV in Europe [11], has developed the National Plan for the Prevention of Viral Hepatitis B and C (PNEV), which includes innovative DAA drugs fund [12]. With the help of DAAs, over 232,000 patients with chronic hepatitis C have been treated, i.e. the highest number of patients treated in Europe [13]. However, the proportion of patients undergoing diagnostic-therapeutic pathways is expected to decrease between 2023 and 2025, along with the increase in the pool of infected but undiagnosed individuals that is currently already high [14]. A study estimated that in 2019 there were 280,000 individuals in Italy with undetected infection and/or not receiving treatments [15]. Without the implementation of a screening program aimed at identifying all infected people, achieving WHO targets will be unlikely [16, 17].

Therefore, in May 2021, the Italian Ministry of Health promoted a regulation for a national program providing free HCV screening tests for specific risk categories, including injecting drug users (IDUs) and prisoners, as well as individuals born between 1969 and 1989. The program included serological tests to search for HCV antibodies and rapid tests, such as capillary test [18]. The national HCV screening test was in an experimental phase until December 2021 [18, 19]. It is worth noting that, alongside the screening strategy, it is necessary to address the issue upstream by increasing the knowledge of this pathology and raising awareness of risk factors, correct behaviors to avoid contagion, early diagnosis, and therapy [20].

Therefore, in this context, it is substantial to explore the population’s knowledge and awareness of this disease, its prevention, and its screening, to identify potential gaps that need to be addressed to bolster the elimination strategy. Thus, just before the beginning of the implementation of the screening program along with awareness campaign in all Italian regions, the present study primarily aimed to explore knowledge about the HCV infection and awareness of the existence of the HCV screening test. The main objective was to assess the level of knowledge regarding the clinical features and treatment of the disease, critical aspects of infection prevention, transmission, and screening test, and to investigate any associated factors. Secondarily, this work aimed to describe attitudes towards this disease and behaviors, such as undergoing HCV test and sharing potentially contaminated items, and to evaluate any associated variables.

## Methods

Between December 2021 and January 2022, a cross-sectional study was conducted in Italy by administering a questionnaire among a sample from the Italian general population. Inclusion criteria for the recruitment were being over 18 years old and understanding the Italian language. The study was conducted in accordance with the Declaration of Helsinki, and the protocol was approved by the Bioethics Committee of the University of Turin. The research was conducted using the Computer-Assisted Web Interview (CAWI) method. A link to access the questionnaire was distributed online on social media (mainly Facebook and Twitter) through the official page of the School of Hygiene and Preventive Medicine of the University of Turin and the profiles of the researchers involved in the study, obtaining a convenience sample. The questionnaire was administered through the online platform LimeSurvey (https://www.limesurvey.org/), provided by the University of Turin. Before accessing the questionnaire, the participants had to accept the informed consent form for data processing and the privacy policy. The questionnaire was voluntary and completely anonymous.

### The Questionnaire

The questionnaire was primarily developed by the researchers based on the available literature. The questionnaire was structured in four sections. The first section collected information about demographics and HCV-related risk factors to identify different risk groups. The three remaining sections aimed at gathering data on knowledge, attitudes, and practices regarding HCV, respectively [2, 21, 22].

The first section collected demographic information such as age, gender and sexual orientation, nationality, region of residency, living condition, presence of children, educational level, and occupation. The occupation specifically explored professionals like healthcare workers, piercers and tattoo artist, barbers, beauticians/chiropodists, i.e. workers at risk for HCV infection [23, 24]. Residency in an urban context or not was explored since for other infectious diseases, e.g. HIV, rural inhabitants were more likely to know less and were at greater risk of infection [25, 26]. Therefore, the number of inhabitants of city of residence of participants was asked, defining urban centers as cities with at least 50,000 inhabitants [27]. This section also collected a few health-related characteristics of both participants and their family members, e.g. chronic diseases, previous HCV test and related result. To identify different risk categories, several specific questions were asked about HCV risk factors, such as history of blood transfusion, reclusion, use of injectable drugs, contact with drug addiction centers, and frequency of using of sexual protection [15].

The second section addressed the level of knowledge by using two scores developed based on scientific literature data [6, 21, 22, 28–30]. The first score was the Disease Knowledge Score, i.e. the first primary outcome of the present study. Notions of basic clinic, symptomatology, and therapy were assessed (12 items, shown in Supplemental Table S1). The Prevention and Transmission Knowledge Score represented the second primary outcome, exploring ways of transmission and preventive behaviors (13 items, shown in Supplemental Table S2). For both Knowledge Scores, both true and false statements were presented and the percentage of correct answers represented the total score. Higher scores corresponded to higher knowledge. Then, participants were asked if they knew about the existence of a screening test for HCV. Being unaware of such test was the last primary outcome. Participants were also asked to select the correct definition of “screening test” to explore their understanding of the topic.

The third section aimed at exploring the attitudes towards HCV infection. The attitude score, i.e. a secondary outcome of this paper, was built out of five different items, about behaviors participants would perform in case of contact with an HCV-positive individual. One point was assigned to each wrong or useless behavior, resulting in a score ranging from 0 to 5 (items listed in Supplemental Table S3). A higher score represented worse/wrong attitudes. Also, the willingness to undergo an HCV test was explored, e.g. participants were asked if they would take a test in case of positive contact and if they would take it if it was administered in association with the COVID-19 test. Participants were asked if they perceived themselves as at risk of contracting HCV and if they were worried about hepatitis C.

In the fourth section, participants were asked if they had ever been tested for HCV (secondary outcome). This section was dedicated to examine different practices related to higher risk of contracting HCV, such as sharing potentially blood-contaminated items (e.g. razors, toothbrush, nail clippers, and earrings) (last secondary outcome). Participants were asked if they were blood donor, had piercings or tattoos, or received aesthetic treatments (e.g. manicure, pedicure, permanent makeup). To better describe the actual risk of exposure to HCV, specific time periods were used to define the answers. For blood donation, 1992 was taken into account: in 1992 the Italian protocols for assessing the eligibility of blood and plasma donors were updated, ensuring the safety and quality of blood donations [31]. For piercings and tattoos, in 1998 the Italian Ministry of Health issued new regulations governing the hygiene and safety of body art practices [24]. In this section, researchers also explored the health educational background, e.g. participants were asked if they personally sought out information and/or if they previously participated in HCV-specific or bloodborne disease health educational programs. Sources of information were explored. Lastly, the participants’ willingness and preferred modality to receive further information on this topic were assessed.

At the end of the online questionnaire, a list of useful websites was displayed to turn the survey into a learning opportunity. A link was also provided to access the European Test Finder, which allows participant to easily access screening test for HCV, HIV and other sexually transmitted diseases.

### Statistical Analysis

All variables underwent descriptive analysis. As the Shapiro-Wilk test indicated non-normal distributions, the median and interquartile range (IQR) were utilized to describe the scalar variables. Chi-square tests and Mann–Whitney tests (Kruskal Wallis where appropriate) were run to assess differences in the distribution of the outcomes across the questionnaire variables.

To explore predictors of the outcomes, multivariable regression models adjusted for age and gender were executed. Linear regressions were used for scalar outcomes (Disease Knowledge Score; Prevention and Transmission Knowledge Score; attitude score) and logistic regressions were used for binary outcomes (being unaware of HCV screening test; having undergone HCV test; sharing blood-contaminated items). The multivariable models were achieved with a stepwise forward selection process, with a univariable p-value < 0.250 as the main criterion [32]. Results were expressed as adjusted Odds Ratios (adjOR) (logistic regression), adjusted Coefficients (adjCoef) (linear regression), and 95% Confidence Intervals (95% CI).

The software SPSS (Statistical Package for Social Science, version 27) and STATA (v16) were used. Missing values were excluded. A p-value less than 0.05 was necessary to deem the results as significant.

## Results

### Characteristics of the Sample

The final sample consisted of 813 participants. Females accounted for 75.1% of the sample. The median age was 33 years (IQR = 28–45). Almost half of the participants (47.1%) had a high school diploma or lower educational level. Details on sociodemographic characteristics are described in Table 1.

**Table 1:** Socio-demographic characteristics of the sample and relationships with primary outcomes

Characteristic	Overall sample	Disease Knowledge Score		Prevention & Transmission Knowledge Score		HCV screening test awareness		
	n = 813 N (%)	Median (IQR)	p	Median (IQR)	p	No n = 173 N (%)	Yes n = 574 N (%)	p
Gender and sexual orientation								
Heterosexual male	156 (20.16)	75 (66.7–83.3)	0.955	46.2 (38.5–53.8)	0.457	24 (16.7)	120 (83.3)	0.343
Heterosexual female	510 (65.9)	75 (66.7–83.3)		46.2 (38.5–53.8)		114 (24.3)	355 (75.7)	
LGBT male	27 (3.5)	66.7 (58.3–75)		46.2 (38.5–53.8)		5 (19.2)	21 (80.8)	
LGBT female	71 (9.2)	75 (66.7–83.3)		46.2 (38.5–46.2)		17 (25.4)	50 (74.6)	
Asexual (both genders)	5 (0.65)	75 (75–75)		46.2 (46.2–46.2)		1 (20.0)	4 (80.0)	
Genderqueer	5 (0.65)	75 (66.7–75)		38.5 (38.5–46.2)		0 (0.0)	5 (100.0)	
Having children								
No	585 (72.0)	75 (66.7–83.3)	0.392	46.2 (38.5–53.8)	0.180	124 (22.9)	417 (77.1)	0.802
Yes	227 (28.0)	75 (66.7–75)		46.2 (38.5–53.8)		49 (23.8)	157 (76.2)	
Living alone								
No	696 (85.6)	75 (66.7–83.3)	0.539	46.2 (38.5–53.8)	0.169	151 (23.7)	487 (76.3)	0.426
Yes	117 (14.4)	75 (66.7–75)		46.2 (38.5–53.8)		22 (20.2)	87 (79.8)	
Place of birth								
Born in Italy with both Italian parents	749 (92.2)	75 (66.7–83.3)	0.499	46.2 (38.5–53.8)	0.318	165 (24.1)	521 (75.9)	**0.006**
Born Abroad	21 (2.6)	75 (66.7–79.2)		46.2 (42.3–53.8)		2 (10.0)	18 (90.0)	
Born in Italy with at least one foreign parent	42 (5.2)	75 (66.7–75)		46.2 (38.5–53.8)		6 (15.0)	34 (85.0)	
Residency								
Northern Italy	544 (66.9)	75 (66.7–83.3)	0.490	46.2 (38.5–53.8)	0.956	128 (25.8)	369 (74.2)	**0.045**
Centre Italy	127 (15.6)	75 (66.7–75)		46.2 (38.5–53.8)		18 (15.5)	98 (84.5)	
Southern Italy	135 (16.6)	75 (66.7–83.3)		46.2 (38.5–53.8)		27 (21.3)	100 (78.7)	
Not in Italy	7 (0.9)	83.3 (66.7–83.3)		46.2 (38.5–53.8)		0 (0.0)	7 (100.0)	
Living in an urban context								
No	481 (59.2)	75 (66.7–79.2)	0.074	46.2 (38.5–53.8)	0.851	110 (24.7)	336 (75.3)	0.235
Yes	332 (40.8)	75 (66.7–83.3)		46.2 (38.5–53.8)		63 (20.9)	238 (79.1)	
Educational level								
High school or lower	383 (47.1)	75 (66.7–75)	**0.009**	46.2 (38.5–53.8)	0.827	83 (24.1)	261 (75.9)	**0.004**
University degree	311 (38.3)	75 (66.7–83.3)		46.2 (38.5–53.8)		77 (26.6)	212 (73.4)	
Masters/Phd	119 (14.6)	75 (66.7–83.3)		46.2 (38.5–53.8)		13 (11.4)	101 (88.6)	
Health care student								
No	697 (85.7)	75 (66.7–75)	**< 0.001**	46.2 (38.5–53.8)	0.097	142 (22.1)	500 (77.9)	0.095
Yes	116 (14.3)	75 (75–83.3)		46.2 (38.5–46.2)		31 (29.5)	74 (70.5)	
Health care worker								
No	682 (83.9)	75 (66.7–75)	**< 0.001**	46.2 (38.5–53.8)	0.255	151 (23.7)	487 (76.3)	0.426
Yes	131 (16.1)	75 (75–83.3)		46.2 (38.5–53.8)		35 (28.7)	87 (71.3)	
Working as tattoo artist, piercer, chiropodist, or barber								
No	791 (97.3)	75 (66.7–83.3)	0.984	46.2 (38.5–53.8)	0.862	169 (23.2)	560 (76.8)	0.924
Yes	22 (2.7)	75 (66.7–75)		46.2 (38.5–53.8)		4 (22.2)	14 (77.8)	

Significant p-values in bold

HCV-related information is fully described in Table 2. Most of the participants (99.4%) were aware of hepatitis C existence, and 4.1% tested positive for HCV. Moreover, 10.5% of the participants reported having at least one family member who tested positive for HCV and 3.2% stated having an HCV-positive partner. Only 7.3% declared they participated in a health education program on HCV.

**Table 2:** HCV-related information and relationships with primary outcomes

Characteristic	Overall sample	Disease knowledge score		Prevention & transmission knowledge score		HCV screening test awareness		
	n = 813 N (%)	Median (IQR)	p	Median (IQR)	p	No n = 173 N (%)	Yes n = 574 N (%)	p
Having heard of hepatitis C before								
No	5 (0.6)	70.9 (58.3–75)	0.283	42.3 (34.6–50)	0.471	1 (25.0)	3 (75.0)	0.930
Yes	808 (99.4)	75 (66.7–83.3)		46.2 (38.5–53.8)		172 (23.1)	571 (76.9)	
Knowing the correct definition of “screening test”								
No	212 (27.6)	66.7 (66.7–75)	**< 0.001**	46.2 (38.5–53.8)	0.484	42 (20.4)	164 (79.6)	0.186
Yes	556 (72.4)	75 (66.7–83.3)		46.2 (38.5–53.8)		131 (24.2)	410 (75.8)	
Subjective perceived risk of contracting HCV infection								
Not at risk	602 (82.2)	75 (66.7–75)	**< 0.001**	46.2 (38.5–53.8)	0.342	134 (22.3)	468 (77.7)	0.183
At risk	130 (17.8)	75 (66.7–83.3)		46.2 (38.5–46.2)		36 (27.7)	94 (72.3)	
Being worried about hepatitis C								
No	617 (84.3)	75 (66.7–83.3)	**< 0.001**	46.2 (38.5–53.8)	0.390	145 (23.5)	472 (76.5)	0.681
Yes	115 (15.7)	66.7 (66.7–75)		46.2 (38.5–53.8)		25 (21.7)	90 (78.3)	
Having being tested for HCV								
No/Don’t know	460 (56.6)	75 (66.7–83.3)	**< 0.001**	46.2 (38.5–53.8)	0.935	101 (23.9)	321 (76.1)	0.568
Yes	353 (43.4)	75 (75-83.3)		46.2 (38.5–53.8)		72 (22.2)	253 (77.8)	
Being HCV positive								
No	773 (95.1)	75 (66.7–83.3)	**0.001**	46.2 (38.5–53.8)	**0.020**	172 (24.2)	539 (75.8)	**0.012**
Yes	33 (4.1)	75 (75-83.3)		38.5 (38.5–46.2)		1 (3.3)	29 (96.7)	
Don’t know	7 (0.9)	75 (58.3–75)		46.2 (38.5–46.2)		0 (0.0)	6 (100.0)	
Being willing to get HCV screening test when getting a COVID-19 rapid test								
No	28 (3.8)	66.7 (58.3–75)	**0.004**	46.2 (30.8–53.8)	0.639	8 (28.6)	20 (71.4)	0.494
Yes	704 (96.2)	75 (66.7–83.3)		46.2 (38.5–53.8)		162 (23.0)	542 (77.0)	
Being willing to undergo HCV screening test in case of positive contact								
No	30 (4.1)	66.7 (58.3–75)	0.126	38.5 (30.8–38.5)	**< 0.001**	8 (26.7)	22 (73.3)	0.648
Yes	702 (95.9)	75 (66.7–83.3)		46.2 (38.5–53.8)		162 (23.1)	540 (76.9)	
Having participated in an HCV prevention program								
No	674 (92.7)	75 (66.7–83.3)	**< 0.001**	46.2 (38.5–53.8)	0.917	160 (23.7)	514 (76.3)	0.420
Yes	53 (7.3)	75 (75-83.3)		46.2 (38.5–46.2)		10 (18.9)	43 (81.1)	
Being interested in receiving more information about HCV								
No	201 (27.6)	75 (66.7–83.3)	0.261	46.2 (38.5–53.8)	0.056	52 (25.9)	149 (74.1)	0.327
Yes	526 (72.4)	75 (66.7–75)		46.2 (38.5–53.8)		118 (22.4)	408 (77.6)	
Having received information about HCV								
Not informed (nether passively nor actively)	388 (53.4)	75 (66.7–83.3)	**< 0.001**	46.2 (38.5–53.8)	0.101	95 (24.5)	293 (75.5)	0.664
Actively informed (± passively informed)	265 (36.5)	75 (75-83.3)		46.2 (38.5–46.2)		57 (21.5)	208 (78.5)	
Only passively informed	74 (10.2)	75 (66.7–83.3)		46.2 (38.5–53.8)		18 (24.3)	56 (75.7)	

Significant p-values in bold

Risk factors and risk behaviors are reported in Table 3. Regarding job categories at risk for HCV infection, 16.1% of participants declared themselves as healthcare professionals, while only 2.7% fell into higher risk professions such as tattoo artists and/or piercers, barbers/hairdressers, beauticians, and chiropodists. A total of 1.5% of the participants reported a history of incarceration. Among the habits that increase the risk of infection, 0.6% reported a history of alcoholism, and 2.3% reported a history of injective drugs. Regarding practices that increase HCV risk, 6.9% underwent transfusions/organ donations, 52.6% received aesthetic treatments, 38.2% had at least one tattoo, and 74.8% had at least one piercing.

**Table 3:** Risk factors, behaviors, and relationships with primary outcomes

Characteristic	Overall sample	Disease knowledge score		Prevention & transmission knowledge score		HCV screening test awareness		
	n = 813 N (%)	Median (IQR)	p	Median (IQR)	p	No n = 173 N (%)	Yes n = 574 N (%)	p
Sharing contaminated items								
No	496 (68.2)	75 (66.7–75)	0.465	46.2 (38.5–53.8)	0.289	112 (22.6)	384 (77.4)	0.453
Yes	231 (31.8)	75 (66.7–83.3)		46.2 (38.5–53.8)		58 (25.1)	173 (74.9)	
Having ever had a sexual intercourse								
No	35 (4.3)	75 (66.7–83.3)	0.881	38.5 (38.5–53.8)	0.032	10 (37.0)	17 (63.0)	0.082
Yes	778 (95.7)	75 (66.7–83.3)		46.2 (38.5–53.8)		163 (22.6)	557 (77.4)	
Frequency of condom use for occasional sex								
Never/almost never	129 (15.9)	75 (66.7–75)	**0.014**	46.2 (38.5–53.8)	0.109	27 (22.9)	91 (77.1)	0.980
Sometimes	62 (7.6)	66.7 (66.7–75)		46.2 (38.5–53.8)		12 (20.7)	46 (79.3)	
Often	98 (12.1)	75 (66.7–83.3)		46.2 (38.5–53.8)		20 (22.2)	70 (77.8)	
Always	139 (17.1)	75 (66.7–83.3)		46.2 (38.5–53.8)		30 (22.7)	102 (77.3)	
Do not have occasional sex	385 (47.4)	75 (66.7–83.3)		46.2 (38.5–53.8)		84 (24.1)	265 (75.9)	
Having a HCV positive partner								
No/Don’t know	787 (96.8)	75 (66.7–83.3)	**0.025**	46.2 (38.5–53.8)	0.674	167 (23.1)	555 (76.9)	0.919
Yes	26 (3.2)	75 (75-83.3)		46.2 (38.5–46.2)		6 (24.0)	19 (76.0)	
Having an addicted partner or housemate								
No/Don’t know	775 (95.3)	75 (66.7–83.3)	0.098	46.2 (38.5–53.8)	0.630	166 (23.3)	546 (76.7)	0.650
Yes	38 (4.7)	75 (75-83.3)		46.2 (38.5–53.8)		7 (20.0)	28 (80.0)	
Having HCV positive family members								
No	670 (82.4)	75 (66.7–83.3)	**0.002**	46.2 (38.5–53.8)	0.665	142 (22.9)	477 (77.1)	**0.005**
Yes	85 (10.5)	75 (66.7–83.3)		46.2 (38.5–53.8)		26 (34.2)	50 (65.8)	
Don’t know	58 (7.1)	66.7 (66.7–75)		46.2 (38.5–53.8)		5 (9.6)	47 (90.4)	
Incarceration personal history								
No	801 (98.5)	75 (66.7–83.3)	0.411	46.2 (38.5–53.8)	0.709	171 (23.2)	565 (76.8)	0.693
Yes	12 (1.5)	75 (75-83.3)		46.2 (30.8–53.8)		2 (18.2)	9 (81.8)	
Alcoholism personal history								
No	808 (99.4)	75 (66.7–83.3)	0.066	46.2 (38.5–53.8)	0.177	173 (23.3)	569 (76.7)	0.218
Yes	5 (0.6)	66.7 (66.7–66.7)		38.5 (30.8–46.2)		0 (0.0)	5 (100.0)	
Being an injective drug user (IDU)								
No	793 (97.7)	75 (66.7–83.3)	0.384	46.2 (38.5–53.8)	0.702	172 (23.6)	556 (76.4)	0.061
Yes	19 (2.3)	75 (66.7–83.3)		46.2 (46.2–46.2)		1 (5.3)	18 (94.7)	
Having ever had an accidental wound caused by a syringe or other instrument contaminated by blood and/or other body fluids								
No	718 (88.3)	75 (66.7–75)	**< 0.001**	46.2 (38.5–53.8)	0.910	151 (22.9)	509 (77.1)	0.617
Yes	95 (11.7)	75 (75-83.3)		46.2 (38.5–46.2)		22 (25.3)	65 (74.7)	
Being a blood donor								
No	527 (64.9)	75 (66.7–83.3)	0.304	46.2 (38.5–53.8)	0.360	119 (24.7)	363 (75.3)	0.181
Yes	285 (35.1)	75 (66.7–83.3)		46.2 (38.5–53.8)		54 (20.4)	211 (79.6)	
Having received blood donation								
No	757 (93.1)	75 (66.7–83.3)	0.969	46.2 (38.5–53.8)	0.652	161 (23.2)	533 (76.8)	0.772
At least one before 1992	16 (2.0)	75 (66.7–83.3)		38.5 (38.5–61.5)		2 (15.4)	11 (84.6)	
Only after 1992	40 (4.9)	75 (66.7–79.2)		46.2 (38.5–53.8)		10 (25)	30 (75)	
Having piercings								
No	205 (25.3)	75 (66.7–83.3)	0.266	46.2 (38.5–53.8)	0.747	41 (21.6)	149 (78.4)	0.224
At least one performed independently	54 (6.7)	75 (66.7–83.3)		46.2 (38.5–53.8)		7 (14)	43 (86)	
At least one performed professionally before 98	248 (30.6)	75 (66.7–75)		46.2 (38.5–53.8)		62 (26.8)	169 (73.2)	
At least one performed professionally after 98	304 (37.5)	75 (66.7–83.3)		46.2 (38.5–53.8)		63 (23)	211 (77)	
Having Tattoos								
No	501 (61.9)	75 (66.7–83.3)	0.149	46.2 (38.5–53.8)	0.372	105 (22.9)	354 (77.1)	0.436
At least one performed not professionally	50 (6.2)	75 (66.7–83.3)		46.2 (38.5–53.8)		10 (21.3)	37 (78.7)	
At least one performed professionally before 1998	15 (1.9)	75 (66.7–75)		46.2 (38.5–53.8)		1 (7.1)	13 (92.9)	
At least one performed professionally after 1998	244 (30.1)	75 (66.7–75)		46.2 (38.5–53.8)		57 (25.3)	168 (74.7)	
Having received an aesthetic treatment								
No	385 (47.4)	75 (66.7–83.3)	0.328	46.2 (38.5–53.8)	0.672	77 (21.8)	277 (78.2)	0.628
Yes	427 (52.6)	75 (66.7–75)		46.2 (38.5–53.8)		95 (24.2)	297 (75.8)	

Significant p-values in bold

The most frequent source of information about HCV was scientific literature (69.4%). Most participants declared they would like to receive information about HCV through brochures (47.5%). (Supplementary Tables: Table S4)

### Primary Outcomes

The median Disease Knowledge Score was 75% (IQR = 66.7–83.3), while the median Prevention and Transmission Knowledge score was 46.2% (IQR = 38.5–53.8). Only 23.2% of participants were unaware of the existence of HCV screening test. Concerning the characteristics of the screening, among those who declared to know its existence, 34.1% thought it measured the severity of symptoms. Descriptive analyses of the items of the knowledge scores and of the variables related to the screening test are available in Supplementary Tables (Tables S1, S2, and S5).

The distribution of the Disease Knowledge Score was significantly different across several variables, such as educational level, belonging to the health sector, having undergone HCV testing, having had an HCV-positive partner or family member, condom frequency of use, having had an accidental injury, the perceived subjective risk of contracting the disease, having participated in an HCV educational program, and having received information on the subject either passively or actively.

As for the Prevention and Transmission Knowledge Score, it was distributed differently across a few variables, such as having HCV and being willing to undergo testing in case of contact with HCV-positive individuals.

Lastly, awareness of the HCV screening test showed significant associations with place of birth, region of residence, educational level, and being or having relatives affected by HCV.

Detailed information about the relationships with the primary outcomes is presented in Tables 1, 2 and 3.

### Secondary Outcomes

The median attitude score was 0 (IQR = 0–1). A total of 43.4% declared that they have undergone HCV test in the past and 31.8% shared potentially blood-contaminated items. Descriptive analyses of the items of the attitude score are available in Supplementary Tables (Tables S3).

The distribution of the Attitude Score was not significantly associated with any of the considered sociodemographic variables. Having undergone an HCV test was significantly associated with having children, living in an urban environment, education, belonging to the health care sector (both workers and students). Sharing potentially blood-contaminated showed significant associations with living alone and belonging to the health care sector (both workers and students). Several variables regarding HCV-related information, risk factors, and behaviors showed significant associations with all secondary outcomes. Detailed information about the relationships with the secondary outcomes is presented in Supplementary Tables (Table S6, S7, and S8).

### Regression Models

#### Primary Outcomes

The multivariable regression models of the primary outcomes are reported in Table 4.

**Table 4:** Regression models of primary outcomes

	Disease knowledge score		Prevention & transmission knowledge score		Not being aware of HCV screening test existence	
	adjCoef (95% CI)	p	adjCoef (95% CI)	p	adjOR (95% CI)	p
Age	− 0.03 (− 0.10; 0.03)	0.306	0.00 (− 0.09; 0.08)	0.929	1.00 (0.99; 1.02)	0.622
Gender and sexual orientation, ref.: heterosexual male						
Heterosexual female	0.74 (− 0.98;2.45)	0.399	− 0.33 (− 2.99; 2.32)	0.807	1.69 (0.82; 3.46)	0.152
LGBT male	− 5.01 (− 8.73; − 1.28)	**0.009**	− 0.64 (− 5.58; 4.29)	0.798	1.13 (0.36; 3.56)	0.835
LGBT female	− 0.39 (− 3.04; 2.26)	0.773	− 1.51 (− 5.22; 2.19)	0.423	1.96 (0.77; 4.95)	0.157
Asexual (both genders)	4.29 (− 3.64; 12.22)	0.289	0.96 (− 9.63; 11.54)	0.859	1.33 (0.13; 13.89)	0.810
Genderqueer	− 2.40 (− 10.23; 5.42)	0.547	− 6.75 (− 17.21; 3.72)	0.206	*	*
Educational level, ref.: high school or lower						
University degree	2.15 (0.62; 3.68)	**0.006**	0.83 (− 1.22; 2.88)	0.426	0.98 (0.64; 1.51)	0.929
Masters/PhD	3.03 (0.98; 5.08)	**0.004**	0.91 (− 1.77; 3.60)	0.504	0.39 (0.19; 0.78)	**0.008**
Living in an urban context, ref.: no						
Yes	0.02 (− 1.35; 1.39)	0.975	–	–	1.01 (0.68; 1.51)	0.953
Living alone, ref.: no						
Yes	–	–	− 1.20 (− 3.72; 1.32)	0.351	–	–
Health care worker, ref.: no						
Yes	3.49 (1.45; 5.54)	**0.001**	− 1.16 (− 3.82; 1.49)	0.390	1.30 (0.74; 2.26)	0.358
Health care student, ref.: no						
Yes	6.25 (3.97; 8.53)	**< 0.001**	− 1.92 (− 4.94; 1.10)	0.212	1.18 (0.45; 2.17)	0.583
Being a blood donor, ref.: no						
Yes	–	–	0.30 (− 1.53; 2.14)	0.747	0.83 (0.55; 1.25)	0.369
Being an injective drug user (IDU), ref.: no						
Yes	–	–	–	–	0.52 (0.05; 5.08)	0.577
Knowing the correct definition of “screening test”, ref.: no						
Yes	–	–	–	–	1.39 (0.88; 2.22)	0.161
Subjective perceived risk of contracting HCV infection, ref.: not at risk						
At risk	–	–	–	–	1.45 (0.87; 2.43)	0.157
Being interested in receiving more information about HCV, ref.: no						
Yes	–	–	1.39 (− 0.62; 3.41)	0.176	–	–
Having a HCV positive partner, ref.: no/don’t know						
Yes	0.62 (− 3.71; 4.95)	0.780	–	–	–	–
Having an addicted partner or housemate, ref.: no/don’t know						
Yes	1.30 (− 2.39; 5.00)	0.489	–	–	–	–
Having ever had an accidental wound caused by a syringe or other contaminated instrument, ref.: no						
Yes	3.48 (1.32; 5.64)	**0.002**	–	–	–	–
Residency, ref.: northern Italy						
Centre Italy	–	–	–	–	0.49 (0.26; 0.90)	**0.022**
Southern Italy	–	–	–	–	0.69 (0.40; 1.19)	0.183
Place of birth, ref.: born in Italy with both Italian parents						
Born Abroad	− 1.06 (− 5.09; 2.97)	0.607	–	–	0.38 (0.08; 1.73)	0.209
Born in Italy with at least one foreign parent	− 2.81 (− 5.62; 0.00)	0.050	–	–	0.60 (0.24; 1.50)	0.273
Having HCV positive family members, ref.: no						
Yes	1.27 (− 0.94; 3.48)	0.260	1.36 (− 1.54; 4.26)	0.357	1.85 (1.04; 3.30)	**0.036**
Don’t know	− 1.52 (− 4.16; 1.12)	0.258	1.156 (− 2.38; 4.69)	0.521	0.45 (0.17; 1.21)	0.115
Being HCV positive, ref.: no						
Yes	6.78 (3.00; 10.56)	**< 0.001**	− 5.49 (− 10.49; − 0.49	**0.031**	0.14 (0.02; 1.25)	0.078
Don’t know	0.58 (− 6.58; 7.74)	0.873	− 3.60 (− 13.08; 5.89)	0.457	*	*
Having piercings, ref.: no						
At least one performed independently	–	–	–	–	0.50 (0.17; 1.53)	0.226
At least one performed professionally before 98	–	–	–	–	0.99 (0.49; 1.98)	0.971
At least one performed professionally after 98	–	–	–	–	0.74 (0.36; 1.52)	0.415
Having tattoos, ref.: no						
At least one performed independently	− 0.81 (− 3.59; 1.98)	0.570	1.58 (2.00; 5.15)	0.387	1.29 (0.55; 3.01)	0.553
At least one performed professionally before 98	2.19 (− 2.53; 6.92)	0.363	− 0.98 (− 7.28; 5.31)	0.759	0.24 (0.03; 1.95)	0.182
At least one performed professionally after 98	− 0.49 (− 2.02; 1.04)	0.530	1.29 (− 0.77; 3.35)	0.220	1.30 (0.83; 2.02)	0.248
Having received an aesthetic treatment, ref.: no						
Yes	–	–	− 0.78 (− 2.86; 1.30)	0.461	–	–
Having received information about HCV, ref.: not informed (nether passively nor actively)						
Actively informed (± passively informed)	4.59 (2.96; 6.22)	**< 0.001**	− 0.53 (− 2.67; 1.59)	0.620	–	–
Only passively informed	2.30 (− 0.004; 4.60)	0.050	0.99 (− 2.07; 4.05)	0.524	–	–

Significant p-values in bold

*Omitted for insufficient sample size

Several variables were significantly associated with the Disease Knowledge Score. University or higher education, health-related study or profession, history of accidental injuries, being affected by HCV and having actively searched for information on HCV were positively associated with this outcome. LGBT males showed significantly lower scores compared with heterosexual males.

Taking into consideration the Prevention and Transmission Knowledge Score only one variable resulted to have a significant association: participants affected by HCV the disease had a negative association with this score.

Regarding being unaware of the existence of the HCV screening test, postgraduate education and living in Central Italy were associated with a lower likelihood of not knowing about the test. Having at least one family member affected by hepatitis C increased the probability of reporting this outcome.

#### Secondary Outcomes

The multivariable regression models of the secondary outcomes are reported in Table 5.

**Table 5:** Regression models of secondary outcomes

	Attitude score		Having being tested for HCV		Sharing contaminated items	
	adjCoef (95% CI)	p	adjOR (95% CI)	p	adjOR (95% CI)	p
Age	0.01 (0.0008; 0.01)	**0.027**	1.00 (0.98; 1.03)	0.925	0.98 (0.96; 1.00)	**0.037**
Gender and sexual orientation, ref.: heterosexual male						
Heterosexual female	− 0.10 (− 0.29; 0.08)	0.277	0.89 (0.42; 1.89)	0.768	0.83 (0.43; 1.59)	0.576
LGBT male	− 0.16 (− 0.46; 0.14)	0.288	2.42 (0.68; 8.69)	0.175	1.30 (0.49; 3.43)	0.595
LGBT female	− 0.13 (− 0.37; 0.11)	0.291	1.55(0.58; 4.11)	0.378	1.40 (0.61; 3.21)	0.423
Asexual (both genders)	0.1 (− 0.53; 0.72)	0.766	1.24 (0.15; 10.22)	0.841	*	*
Genderqueer	− 0.32 (− 0.96; 0.32)	0.325	1.78 (0.18; 18.12)	0.625	0.79 (0.06; 7.61)	0.753
Educational level, ref.: high school or lower						
University degree	0.02 (− 0.1; 0.15)	0.698	0.96 (0.59; 1.57)	0.885	1.05 (0.70; 1.57)	0.810
Masters/PhD	0.03 (− 0.14; 0.20)	0.722	1.78 (0.93; 3.41)	0.082	0.91 (0.53; 1.57)	0.764
Living in an urban context, ref.: no						
Yes	− 0.07 (− 0.18; 0.04)	0.205	1.61 (1.05; 2.48)	**0.029**	1.26 (0.88; 1.81)	0.203
Living alone, ref.: no						
Yes	–	–	–	–	0.27 (0.15; 0.51)	**< 0.001**
Having children, ref.: no						
Yes	− 0.01 (− 0.15; 0.14)	0.934	2.34 (1.28; 4.28)	**0.006**	–	–
Health care worker, ref.: no						
Yes	0.08 (− 0.09; 0.25)	0.382	2.41 (1.27; 4.58)	**0.007**	1.61 (0.96; 2.70)	0.071
Working as tattoo artist, piercer, chiropodist, or barber, ref.: no						
Yes	–	–	–	–	0.25 (0.05; 1.19)	0.082
Health care student, ref.: no						
Yes	0.01 (− 0.19; 0.18)	0.941	1.47 (0.75; 2.88)	0.262	1.32 (0.75; 2.34)	0.336
Incarceration personal history, ref.: no						
Yes	− 0.42 (− 0.96; 0.12)	0.124	–	–	–	–
Being a blood donor, ref.: no						
Yes	− 0.14 (− 0.25; − 0.03)	**0.013**	1.93 (1.25; 2.95)	**0.003**	1.13 (0.79; 1.63)	0.491
Having received blood donation, ref.: no						
At least one before 1992	–	–	5.32 (1.13; 25.13)	**0.035**	–	–
Only after 1992	–	–	1.89 (0.77; 4.65)	0.167	–	–
Being an injective drug user (IDU), ref.: no						
Yes	− 0.11 (− 0.56; 0.33)	0.621	–	–	–	–
Subjective perceived risk of contracting HCV infection, ref.: not at risk						
At risk	− 0.09 (− 0.24; 0.07)	0.268	2.56 (1.39; 4.72)	0.003	1.03 (0.64; 1.67)	0.896
Being worried about hepatitis C, ref.: no						
Yes	–	–	0.62 (0.33; 1.17)	0.140	–	–
Being interested in receiving more information about HCV, ref.: no						
Yes	− 0.12 (− 0.24; 0.00)	0.054	–	–	–	–
Having a HCV positive partner, ref.: no/don’t know						
Yes	− 0.13 (− 0.47; 0.22)	0.473	20.93 (2.13; 205.7)	**0.009**	0.61 (0.17; 2.12)	0.435
Having an addicted partner or housemate, ref.: no/don’t know						
Yes	0.04 (− 0.28; 0.36)	0.803	1.28 (0.39; 4.22)	0.689	–	–
Number of partners	–	–	1.04 (0.96; 1.13)	0.351	–	–
Frequency of condom use for occasional sex, ref.: never/almost never						
Sometimes	–	–	1.56 (0.61; 4.00)	0.358	–	–
Often	–	–	2.49 (1.10; 5.65)	**0.029**	–	–
Always	–	–	2.02 (0.96; 4.28)	0.065	–	–
Do not have occasional sex	–	–	2.18 (1.14; 4.19)	**0.019**	–	–
Having ever had an accidental wound caused by a syringe or other contaminated instrument, ref.: no						
Yes	− 0.11 (− 0.29; 0.06)	0.212	4.11 (1.92; 8.83)	**< 0.001**	–	–
Place of birth, ref.: born in Italy with both Italian parents						
Born Abroad	–	–	3.24 (0.85; 12.42)	0.086	–	–
Born in Italy with at least one foreign parent	–	–	1.09 (0.43; 2.78)	0.850	–	–
Residency, ref.: northern Italy						
Centre Italy	− 0.03 (− 0.18; 0.11)	0.650	–	–	–	–
Southern Italy	0.12 (− 0.03; 0.26)	0.121	–	–	–	–
Not in Italy	− 0.11 (− 0.64; 0.42)	0.684	–	–	–	–
Having HCV positive family members, ref.: no						
Yes	− 0.17 (− 0.35; 0.00)	0.055	1.85 (0.95; 3.61)	0.069	–	–
Don’t know	0.01 (− 0.20; 0.22)	0.944	1.01 (0.40; 2.53)	0.982		
Being HCV positive, ref.: no						
Yes	0.08 (− 0.28; 0.43)	0.669	–	–	0.10 (0.01; 0.79)	**0.030**
Don’t know	− 0.56 (− 1.15; 0.02)	0.057	–	–	1.46 (0.23; 9.19)	0.684
Having Piercings, ref.: no						
At least one performed independently	− 0.05 (− 0.32; 0.23)	0.734	0.96 (0.30; 3.03)	0.940	1.85 (0.73; 4.69)	0.193
At least one performed professionally before 98	− 0.02 (0.21; 0.17)	0.849	1.48 (0.68; 3.20)	0.324	1.78 (0.90; 3.52)	0.098
At least one performed professionally after 98	0.09 (− 0.10; 0.29)	0.360	1.36 (0.62; 3.00)	0.448	1.19 (0.61; 2.32)	0.615
Having Tattoos, ref.: no						
At least one performed independently	0.18 (− 0.06; 0.42)	0.133	2.28 (0.87; 5.94)	0.093	1.63 (0.78; 3.43)	0.194
At least one performed professionally before 98	− 0.08 (− 0.47; 0.30)	0.668	2.28 (0.51; 10.17)	0.283	1.56 (0.42; 5.85)	0.509
At least one performed professionally after 98	− 0.06 (− 0.18; 0.07)	0.373	0.89 (0.55; 1.46)	0.658	1.19 (0.80; 1.78)	0.384
Having received information about HCV, ref.: not informed (nether passively nor actively)						
Actively informed (± passively informed)	− 0.11 (− 0.24; 0.03)	0.110	6.61 (4.10; 10.68)	**< 0.001**	–	–
Only passively informed	− 0.13 (− 0.31; 0.06)	0.173	5.25 (2.69; 10.23)	**< 0.001**	–	–
Being willing to undergo HCV screening test in case of positive contact, ref.: no						
Yes	–	–	2.09 (0.63; 7.01)	0.231	3.72 (1.06; 13.11)	**0.041**
Disease Knowledge Score	− 0.01 (− 0.01; − 0.00002)	**0.049**	1.03 (1.01; 1.06)	**0.009**	1.00 (0.98; 1.02)	0.971
Prevention & Transmission Knowledge Score	0.01 (0.003; 0.01)	**0.001**	1.00 (0.98; 1.02)	0.883	1.00 (0.99; 1.02)	0.859
Attitude score	–	–	0.86 (0.63; 1.17)	0.337	0.91 (0.70; 1.18)	0.484

Significant p-values in bold

*Omitted for insufficient sample size

Regarding the attitude score, the Disease Knowledge Score and being a blood donor showed negative association with the outcome, while the Prevention and Transmission Knowledge score and age showed positive association.

Considering the previous execution of the HCV test, there was a higher likelihood of being a health care worker, living in an urban context, having children, having donated blood or received blood transfusions before 1992, perceiving subjective HCV risk, often wearing protection during sex, not having occasional sexual intercourses, having an HCV-positive partner, and being informed on HCV. A higher Disease Knowledge Score was associated with a higher probability of reporting this outcome.

Age, living alone, and being affected by HCV were associated with a lower likelihood of sharing potentially contaminated items, while being willing to undergo HCV screening test in case of positive contact showed a higher likelihood.

## Discussion

The present study primarily aimed to assess HCV-related knowledge (both about the disease and its prevention and transmission) and awareness about the existence of HCV screening test and to explore potentially associated factors. Secondarily, this work aimed to evaluate attitudes towards this disease and behaviors, such as undergoing HCV test and sharing potentially contaminated items, analyzing which factors may influence these outcomes.

First, it is worth noting that the percentages of right answers were higher for the Disease Knowledge Score (75%) than for the Prevention and Transmission Knowledge Score (46%). While these findings highlight a fair knowledge on the disease, the knowledge about prevention and transmission is poor and alarming from a public health perspective. This result could direct the efforts of awareness campaigns towards this gap. The gap between the two aforementioned types of knowledge is not new; for instance, Zainiddinov and colleagues highlighted a similar scenario concerning HIV [33].

Concerning the potential predictors of the two Knowledge Scores in the multivariable models, several considerations should be made. HCV-positive patients reported significant associations with both outcomes, but in opposite directions. On the one hand, these participants were more aware of the features and complications of the disease; on the other hand, they were less likely to be informed on how to transmit the infection. Hence, although the information conveyed to patients (likely by their treating physician) appeared to effectively increase awareness of the clinical implications and severity, it did not seem to enhance the knowledge necessary to substantially reduce transmission. The association between knowledge about the disease and having such disease has also been reported for sexual transmitted diseases, such as HIV [34]. Interestingly, no other variables were associated with the Prevention and Transmission Knowledge score, suggesting that even the categories of the population that are well-informed about HCV need to receive specific education on prevention and transmission, which are the most crucial topics to reduce the spread. Regarding the categories of participants who are better informed about the disease, the findings of the present paper showed significant associations for highly educated participants, healthcare workers and students, participants who actively sought information, and individuals who have had accidental injuries with blood-contaminated items. The relationship between higher levels of education and higher disease knowledge has already been described for HCV [35, 36] and for many other conditions, such as HIV and other sexually transmitted diseases [36–39]. Similarly, it is not surprising to find a positive association between the Disease Knowledge Score and being healthcare students/workers, who represent a specific population with, in general, higher health literacy [40, 41], or with participants who have had accidental injuries, as they may have received information about the possible risks related to the injury. Also individuals who actively searched for information on HCV were more likely to score high on the Disease Knowledge score. This is particularly relevant considering the Information-Motivation-Behavioral Skills model proposed by Fisher and colleagues for HIV preventive behavior [42]. Indeed, both information and motivation are essential in engaging preventive behaviors: participants with high knowledge and high interest in searching for information might represent a specific group of people who are more prone to change their behaviors. However, the fact that there was no association between these above-mentioned categories and a higher Prevention and Transmission knowledge was unexpected and may represent a relevant issue that should be further investigated, especially considering healthcare worker education and information that can be found through active searching.

The current study has brought attention to male LGBT + individuals as a vulnerable group with limited Disease Knowledge. This indicates an urgent need for awareness campaigns targeted specifically towards this population. Indeed, men who have sex with men are known to have a higher burden of hepatitis and other sexually transmitted diseases than the general population [43].

With regards to the third outcome, the majority of participants demonstrated awareness of the HCV screening test. This implies that even though information campaigns about the screening program were not extensively carried out in most regions of Italy during the study period, participants were knowledgeable (or at least had an idea) about the existence of the test. Future studies on the Italian population should compare these results with data collected after the implementation of awareness campaigns aimed at incentivizing participation in the HCV screening program. Participants with higher education reported greater awareness, consistent with the previously discussed findings related to knowledge about the disease, as well as those residing in Central Italy. The latter result could potentially be attributed to the possibility that awareness campaigns started earlier in certain regions of Central Italy. A comparative analysis of HCV screening test planning at the regional level could reveal best practices and serve as a model for developing more effective health promotion plans. It is noteworthy that individuals with HCV-positive family members had a lower level of awareness regarding the screening test, indicating the need for more comprehensive information dissemination not only to HCV-positive patients but also to those in their immediate circles.

The study of secondary outcomes (i.e. attitude score, having undergone HCV test, and sharing blood-contaminated items) highlighted some relevant issues. First, attitudes in case of contact with an HCV-positive individual were quite good. Interestingly, while high disease knowledge was associated with better attitudes as one would expect, high prevention and transmission knowledge was associated with worse/wrong attitudes. This could happen for several reason related to the perception towards the disease. Perhaps, if someone has a high level of knowledge about how HCV is transmitted, they may overestimate the risk of contracting the virus through casual contact with an infected person. This could lead to unnecessary avoidance or fear of HCV-positive individuals. Indeed, despite advances in understanding HCV and how it is spread, there is still a lot of stigma surrounding the virus. People who have a high level of knowledge about HCV transmission may be more likely to hold stigmatizing attitudes towards HCV-positive individuals, as seen, for instance, among healthcare professionals [44, 45].

The role of risk perception was also relevant with regard to the second secondary outcome, which was having been tested for HCV. First, it should be noted that our sample reported a quite higher percentage of people tested for HCV compared with other Italian data on university students [46]; however, differences in sample composition probably explain this result. In terms of risk perception, several items related to an overall high risk perception and a high interest towards the disease (such as a high subjective perceived risk of contracting HCV infection, wearing condoms or not having occasional sex, and being informed about HCV) were associated with a higher likelihood of having been tested. Overall, individuals who have a higher overall risk perception of HCV may be more likely to get tested for the virus due to their motivation to protect their health, awareness of the potential consequences of infection (as shown by the relationship between Disease Knowledge Score and this outcome), and increased information-seeking behavior. Some authors have reported that people who underestimate the infection attach less value to medical monitoring [46–48]. Not surprisingly, certain groups may have a higher likelihood of being tested for HCV, due to their increased risk of exposure to the virus (i.e. healthcare workers, people with children, blood donors, blood recipients, and individuals with a positive partner). For instance, healthcare workers may have access to routine testing as part of their job, blood donors and recipients are often screened for HCV as a precautionary measure. Interestingly, urban populations had a higher likelihood of being tested for HCV, probably due to better access to healthcare services and fewer stigmatizing encounters with healthcare professionals compared with rural populations [49–51]. Considering the last secondary outcome, a substantial percentage of the sample usually shared potentially blood-contaminated items, indicating that most people are not concerned about the objects they use in their daily lives, but they probably see them as less dangerous tools than those more closely associated with infections in the common imagination, such as syringes. It is worth noting that, despite not showing higher levels of knowledge regarding prevention and transmission, HCV-positive individuals have shown to share these objects less frequently, suggesting that they have been informed about the appropriate behaviors to follow and demonstrate good habits.

The present study had some limitations. First, using a cross-sectional design allows hypothesis formulation, but not causal relationships identification between predictors and outcomes. Another limitation was the opportunistic sampling and the use of social media invites for the questionnaire, which limited participation to those who are registered. Finally, it is not possible to determine if and where the awareness campaigns had already been initiated.

In conclusion, this study provided important insights into the knowledge and awareness of HCV among a sample from the general population in Italy. The results highlighted a concerning lack of knowledge about prevention and transmission, indicating a need for targeted education campaigns to bridge this gap. The findings also emphasized the importance of information and motivation, indicating the need for comprehensive and easily accessible information sources. Moreover, the study identified LGBT + men as a vulnerable group with limited disease knowledge, underlining the need for targeted awareness campaigns. Overall, the study’s findings have important implications for public health campaigns aimed at reducing the spread of HCV. It is vital to improve knowledge and awareness of prevention and transmission among the general population, as well as to provide comprehensive information to vulnerable groups. The study’s results could guide future research and inform the development of effective health promotion plans. Another important area of future research could concern the effectiveness of awareness campaigns on participation in HCV screening programs. By comparing the results of this study with those of a potential future study conducted after the implementation of specific information campaigns on the subject, the effectiveness and impact of such campaigns could be evaluated.

## Supplementary Information

Below is the link to the electronic supplementary material.

Supplementary material 1 (PDF 352.3 kb)

## Data Availability

The datasets generated during and/or analysed during the current study are available from the corresponding author on reasonable request.
