# Supplementary material for: Exploring Knowledge and Awareness of HCV Infection and Screening Test: A Cross-Sectional Survey Among an Italian Sample
Source: J Community Health. 2023 Apr 28;48(5):769–83. doi: 10.1007/s10900-023-01218-4 (PMC10144876; doi:10.1007/s10900-023-01218-4)
Supplement: Supplementary file 1 — Supplementary material 1 (PDF 352.3 kb) [file 10900_2023_1218_MOESM1_ESM.pdf]

# Supplementary Tables

**Title:**

**Exploring knowledge and awareness of HCV infection and screening test in Italy: a nationwide survey**


**Authors:**

Giuseppina Lo Moro[1], Giacomo Scaioli[1,2], Lorenzo Vola[1], Laura Guastavigna[1], Roberta Frattin[1], Elisabetta De Vito[3], Fabrizio Bert[1,2]*, Roberta Siliquini[1,4]

**Affiliations:**

[1] Department of Public Health Sciences and Pediatrics, University of Turin, Turin, Italy

[2] Health Local Unit "ASL TO3", Turin, Italy

[3] Department of Human, Social and Health Sciences, University of Cassino and Southern Lazio, Cassino, Italy

[4] A.O.U. City of Health and Science of Turin, Turin, Italy

***Corresponding author: Fabrizio Bert fabrizio.bert@unito.it**


Table of contents


Table S1. Items of the Disease Knowledge score ........ 2

Table S2. Items of the Prevention & Transmission Knowledge Score ........ 4

Table S3. Items of the attitudes score ........ 6

Table S4. Sources of information about HCV ........ 7

Table S5. Screening test related variables (completed only by participants who declared to know about the existence of a screening test) ........ 8

Table S6. Socio-demographic characteristics of the sample and relationships with secondary outcomes... 9

Table S7. HCV-related information and relationships with secondary outcomes ........ 11

Table S8. Risk factors, behaviors, and relationships with secondary outcomes ........ 13

**Table S1. Items of the Disease Knowledge score**

| Variable | Frequency (N) | Percentage (%) |
|---|---|---|
| **HCV is a chronic disease** | | |
| No | 7 | 0.9 |
| Yes | 580 | 75.3 |
| Don't know | 183 | 23.8 |
| **Percentage of HCV chronicity** ***(not included in the Disease Knowledge score)*** | | |
| < 25% | 69 | 11.9 |
| 26-54% | 145 | 25.0 |
| 55-85% | 215 | 37.1 |
| 86-95% | 94 | 16.2 |
| > 95% | 56 | 9.7 |
| **Can a healthy looking subject be HCV positive?** | | |
| No | 22 | 2.9 |
| Yes | 748 | 97.1 |
| **Most affected organ by HCV** | | |
| Heart | 4 | 0.5 |
| Bones | 2 | 0.3 |
| Liver | 745 | 96.8 |
| Stomach | 2 | 0.3 |
| Pancreas | 14 | 1.8 |
| Lung | 3 | 0.4 |
| **Can HCV cause asthma?** | | |
| No | 763 | 99.0 |
| Yes | 8 | 1.0 |
| **Can HCV cause heart disease?** | | |
| No | 696 | 90.3 |
| Yes | 75 | 9.7 |
| **Can HCV cause hepatic cirrhosis?** | | |
| No | 95 | 12.3 |
| Yes | 676 | 87.7 |
| **Can HCV cause diabetes?** | | |

| | | |
|---|---|---|
| No | 700 | 90.8 |
| Yes | 71 | 9.2 |
| **Can HCV cause rupture of the spleen?** | | |
| No | 691 | 89.6 |
| Yes | 80 | 10.4 |
| **Can HCV cause scarlet fever?** | | |
| No | 767 | 99.5 |
| Yes | 4 | 0.5 |
| **Can HCV cause Down Syndrome?** | | |
| No | 771 | 100.0 |
| Yes | 0 | 0 |
| **Can HCV cause hepatocellular cancer?** | | |
| No | 227 | 29.4 |
| Yes | 544 | 70.6 |
| **Can HCV be cured with drugs?** | | |
| No, it’s not curable | 99 | 12.9 |
| Healing is complete in most subjects | 299 | 38.8 |
| Healing is not complete and there is a high risk of reinfection | 372 | 48.3 |

**Table S2. Items of the Prevention & Transmission Knowledge Score**

| Variable | Frequency (N) | Percentage (%) |
|---|---|---|
| **Can HCV be transmitted with a kiss?** | | |
| No | 653 | 84.9 |
| Yes | 116 | 15.1 |
| **Can HCV be transmitted by sharing the toothbrush?** | | |
| No | 423 | 55.0 |
| Yes | 346 | 45.0 |
| **Can HCV be transmitted by using water and contaminated food prepared by HCV infected people?** | | |
| No | 650 | 84.5 |
| Yes | 119 | 15.5 |
| **Can vaccines transmit HCV?** | | |
| No | 704 | 91.5 |
| Yes | 65 | 8.5 |
| **Can HCV be transmitted by using public toilets?** | | |
| No | 629 | 81.8 |
| Yes | 140 | 18.2 |
| **Can not sterilized syringes, needles and surgical instruments transmit HCV?** | | |
| No | 8 | 1.0 |
| Yes | 761 | 99.0 |
| **Can HCV be transmitted by getting a tattoo/piercing?** | | |
| No | 52 | 6.8 |
| Yes | 717 | 93.2 |
| **Can insect sting transmit HCV?** | | |
| No | 658 | 85.6 |
| Yes | 111 | 14.4 |
| **Can HCV be transmitted by during pregnancy or birth in case of positive mother?** | | |
| No | 139 | 18.1 |
| Yes | 629 | 81.9 |
| **Can you be infected by HCV standing in the same room with person HCV positive for more than 15 minutes without a mask?** | | |
| No | 761 | 99.1 |

| | | |
|---|---|---|
| Yes | 7 | 0.9 |
| **Can HCV be transmitted by through wounds / open cuts?** | | |
| No | 57 | 7.4 |
| Yes | 712 | 92.6 |
| **Can HCV be prevented with a vaccine?** | | |
| No | 579 | 75.4 |
| Yes | 189 | 24.6 |
| **Can HCV be prevented by avoiding risky behavior?** | | |
| No | 11 | 1.4 |
| Yes | 758 | 98.6 |

**Table S3. Items of the attitudes score**

| Variable | Frequency (N) | Percentage (%) |
|---|---|---|
| **Shaking hands with an HCV affected person** | | |
| No | 34 | 4.6 |
| Yes | 698 | 95.4 |
| **Using condoms with an HCV affected person** | | |
| No | 293 | 40.0 |
| Yes | 439 | 60.0 |
| **Never using condoms with an HCV affected person** | | |
| No | 720 | 98.4 |
| Yes | 12 | 1.6 |
| **Sharing personal belonging (toothbrush, razor, nail clipper, earrings) with an HCV affected person** | | |
| No | 707 | 96.6 |
| Yes | 25 | 3.4 |
| **Wearing a mask in presence of an HCV affected person** | | |
| No | 677 | 92.5 |
| Yes | 55 | 7.5 |

**Table S4. Sources of information about HCV**

| Variable* | Frequency (N) | Percentage (%) |
|---|---|---|
| **Modalities through which the participant has been informed about HCV** | | |
| Magazines/journals | 115 | 43.4 |
| Ministry sites | 80 | 30.2 |
| Scientific literature | 184 | 69.4 |
| Territorial health service/General practitioner/Medical office | 88 | 33.2 |
| Friends | 26 | 9.8 |
| Internet/social media | 17 | 6.4 |
| Passive information at school/university | 59 | 66.3 |
| Passive information by the web | 16 | 18.0 |
| Passive information by the television | 10 | 11.2 |
| Passive information through friends | 24 | 27.0 |
| Passive information randomly | 16 | 18.0 |
| **Modalities through which the participant would like to receive information about HCV:** | | |
| Short video | 208 | 39.5 |
| Brochure | 250 | 47.5 |
| Website with certified information | 152 | 28.9 |
| Educational event in the Territorial health service | 109 | 20.7 |
| Dialogue with your general practitioner | 112 | 21.3 |
| Scientific television program | 128 | 24.3 |
| Occasional advertising | 91 | 17.3 |
| Social media (facebook; twitter; instagram; tiktok) | 158 | 30.0 |
| Educational event during working hours | 107 | 20.3 |

***Only "yes" answers are presented in the table, and "no" answers are omitted**

**Table S5. Screening test related variables (completed only by participants who declared to know about the existence of a screening test)**

| Variable | | Frequency (N) | Percentage (%) |
|---|---|---|---|
| **Is the HCV screening test mandatory?** | | | |
| No | | 565 | 98.4 |
| Yes | | 9 | 1.6 |
| **Is the HCV screening a free-of-charge test (if you are in a class with risk of infection)?** | | | |
| No | | 82 | 14.3 |
| Yes | | 492 | 85.7 |
| **Does HCV screening test influence the progression of HCV related disease?** | | | |
| No | | 269 | 46.9 |
| Yes | | 304 | 53.1 |
| **Does HCV screening test measure the severity of symptoms associated with the pathology?** | | | |
| No | | 377 | 65.9 |
| Yes | | 195 | 34.1 |

**Table S6. Socio-demographic characteristics of the sample and relationships with secondary outcomes**

| Characteristic | Attitude Score | | Having being tested for HCV | | | Sharing contaminated items | | |
|---|---|---|---|---|---|---|---|---|
| | **Median (IQR)** | **p** | **NO n=460 N (%)** | **YES n=353 N (%)** | **p** | **NO n=496 N (%)** | **YES n=231 N (%)** | **p** |
| **Gender and sexual orientation** | | | | | | | | |
| Heterosexual male | 0 (0-1) | 0.302 | 87 (55.8) | 69 (44.2) | 0.348 | 104 (73.8) | 37 (26.2) | 0.123 |
| Heterosexual female | 0 (0-1) | | 301 (59.0) | 209 (40.9) | | 312 (68.6) | 143 (31.4) | |
| LGBT male | 0 (0-1) | | 13 (48.1) | 14 (51.8) | | 16 (61.5) | 10 (38.5) | |
| LGBT female | 0 (0-1) | | 33 (46.5) | 38 (53.5) | | 38 (57.6) | 28 (42.4) | |
| Asexual (both genders) | 0 (0-1) | | 3 (60.0) | 2 (40.0) | | 5 (100.0) | 0 (0.0) | |
| Genderqueer | 0 (0-0) | | 2 (40.0) | 3 (60.0) | | 4 (80.0) | 1 (20.0) | |
| **Having children** | | | | | | | | |
| No | 0 (0-1) | 0.68 | 344 (58.8) | 241 (41.2) | 0.036 | 359 (68.3) | 167 (31.7) | 0.981 |
| Yes | 0 (0-1) | | 115 (50.7) | 112 (49.3) | | 137 (68.2) | 64 (31.8) | |
| **Living alone** | | | | | | | | |
| No | 0 (0-1) | 0.933 | 394 (56.6) | 302 (43.4) | 0.968 | 406 (65.3) | 216 (34.7) | <0.001 |
| Yes | 0 (0-1) | | 66 (56.4) | 51 (43.6) | | 90 (85.7) | 15 (14.3) | |
| **Place of birth** | | | | | | | | |
| Born in Italy with both Italian parents | 0 (0-1) | 0.983 | 428 (57.1) | 321 (42.9) | 0.088 | 455 (68.2) | 212 (31.8) | 0.530 |
| Born Abroad | 0 (0-1) | | 7 (33.3) | 14 (66.7) | | 11 (57.9) | 8 (42.1) | |
| Born in Italy with at least one foreign parent | 0.5 (0-1) | | 25 (59.5) | 17 (40.5) | | 29 (72.5) | 11 (27.5) | |
| **Residency** | | | | | | | | |
| Northern Italy | 0 (0-1) | 0.210 | 305 (56.1) | 239 (43.9) | 0.603 | 327 (67.6) | 157 (32.4) | 0.749 |
| Centre Italy | 0 (0-1) | | 68 (53.5) | 59 (46.5) | | 82 (71.9) | 32 (28.1) | |
| Southern Italy | 1 (0-1) | | 83 (61.5) | 52 (38.5) | | 83 (68) | 39 (32) | |
| Not in Italy | 0 (0-1) | | 4 (57.1) | 3 (42.9) | | 4 (57.1) | 3 (42.9) | |
| **Living in an urban context** | | | | | | | | |
| No | 0 (0-1) | 0.488 | 291 (60.5) | 190 (39.5) | 0.007 | 308 (70.5) | 129 (29.5) | 0.109 |
| Yes | 0 (0-1) | | 169 (50.9) | 163 (49.1) | | 188 (64.8) | 102 (35.2) | |
| **Educational level** | | | | | | | | |
| High school or lower | 0 (0-1) | 0.961 | 224 (58.5) | 159 (41.5) | 0.075 | 234 (70.1) | 100 (29.9) | |

| | | | | | | | | |
|---|---|---|---|---|---|---|---|---|
| University degree | 0 (0-1) | | 180 (57.9) | 131 (42.1) | | 186 (65.5) | 98 (34.5) | 0.447 |
| Masters/Phd | 0 (0-1) | | 56 (47.1) | 63 (52.9) | | 76 (69.7) | 33 (30.3) | |
| **Health care student** | | | | | | | | |
| No | 0 (0-1) | 0.092 | 407 (58.4) | 290 (41.6) | 0.011 | 437 (69.9) | 188 (30.1) | 0.015 |
| Yes | 0 (0-1) | | 53 (45.7) | 63 (54.3) | | 59 (57.8) | 43 (42.2) | |
| **Health care worker** | | | | | | | | |
| No | 0 (0-1) | 0.841 | 427 (62.6) | 255 (37.4) | <0.001 | 425 (69.8) | 184 (30.2) | 0.040 |
| Yes | 0 (0-1) | | 33 (25.2) | 98 (74.8) | | 71 (60.2) | 47 (39.8) | |
| **Working as tattoo artist, piercer, chiropodist, or barber** | | | | | | | | |
| No | 0 (0-1) | 0.618 | 450 (56.9) | 341 (43.1) | 0.286 | 480 (67.7) | 229 (32.3) | 0.057 |
| Yes | 0 (0-1) | | 10 (45.5) | 12 (54.5) | | 16 (88.9) | 2 (11.1) | |

**Table S7. HCV-related information and relationships with secondary outcomes**

| Characteristic | Attitude Score | | Having being tested for HCV | | | Sharing contaminated items | | |
|---|---|---|---|---|---|---|---|---|
| | Median (IQR) | p | NO n=460 N (%) | YES n=353 N (%) | p | NO n=496 N (%) | YES n=231 N (%) | p |
| **Having heard of hepatitis C before** | | | | | | | | |
| No | 1 (1-1.5) | 0.029 | 3 (60.0) | 2 (40.0) | 0.877 | 3 (100.0) | 0 (0.0) | 0.236 |
| Yes | 0 (0-1) | | 457 (56.6) | 351 (43.4) | | 493 (68.1) | 231 (31.9) | |
| **Knowing the correct definition of "Screening test"** | | | | | | | | |
| No | 1 (0-1) | 0.014 | 149 (70.3) | 63 (29.7) | <0.001 | 149 (75.6) | 48 (24.4) | 0.009 |
| Yes | 0 (0-1) | | 285 (51.3) | 271 (48.7) | | 347 (65.5) | 183 (34.5) | |
| **Subjective perceived risk of contracting HCV infection** | | | | | | | | |
| Not at risk | 0 (0-1) | 0.005 | 381 (63.3) | 221 (36.7) | <0.001 | 414 (69.2) | 184 (30.8) | 0.210 |
| At risk | 0 (0-1) | | 31 (23.8) | 99 (76.2) | | 82 (63.6) | 47 (36.4) | |
| **Being worried about hepatitis C** | | | | | | | | |
| No | 0 (0-1) | 0.059 | 326 (52.8) | 291 (47.2) | <0.001 | 414 (67.4) | 200 (32.6) | 0.281 |
| Yes | 1 (0.5-1.5) | | 86 (74.8) | 29 (25.2) | | 82 (72.6) | 31 (27.4) | |
| **Having being tested for HCV** | | | | | | | | |
| No/Don't know | 1 (0-1) | <0.001 | // | // | // | 280 (68.3) | 130 (31.7) | 0.965 |
| Yes | 0 (0-1) | | // | // | | 216 (68.1) | 101 (31.9) | |
| **Being HCV positive** | | | | | | | | |
| No | 0 (0-1) | 0.030 | 455 (58.9) | 318 (41.1) | <0.001 | 463 (67.0) | 228 (33.0) | 0.003 |
| Yes | 0 (0-1) | | 0 (0.0) | 33 (100.0) | | 29 (96.7) | 1 (3.3) | |
| Don't know | 0 (0-0) | | 5 (71.4) | 2 (28.6) | | 4 (66.7) | 2 (33.3) | |
| **Being willing to get HCV screening test when getting a COVID-19 rapid test** | | | | | | | | |
| No | 1 (1-1) | <0.001 | 19 (67.9) | 9 (32.1) | 0.208 | 23 (82.1) | 5 (17.9) | 0.107 |
| Yes | 0 (0-1) | | 393 (55.8) | 311 (44.2) | | 473 (67.7) | 226 (32.3) | |
| **Being willing to undergo HCV screening test in case of positive contact** | | | | | | | | |
| No | 1 (0-1) | 0.217 | 21 (70.0) | 9 (30.0) | 0.122 | 27 (90.0) | 3 (10.0) | 0.009 |
| Yes | 0 (0-1) | | 391 (55.7) | 311 (44.3) | | 469 (67.3) | 228 (32.7) | |
| **Having participated in an HCV prevention program** | | | | | | | | |
| No | 0 (0-1) | 0.061 | 399 (59.2) | 275 (40.8) | <0.001 | 459 (68.1) | 215 (31.9) | 0.797 |
| Yes | 0 (0-1) | | 11 (20.8) | 42 (79.2) | | 37 (69.8) | 16 (30.2) | |
| **Being interested in receiving more information about HCV** | | | | | | | | |

| | | | | | | | | |
|---|---|---|---|---|---|---|---|---|
| No | 1 (0-1) | 0.05 | 98 (48.8) | 103 (51.2) | 0.010 | 146 (72.6) | 55 (27.4) | 0.114 |
| Yes | 0 (0-1) | | 312 (59.3) | 214 (40.7) | | 350 (66.5) | 176 (33.5) | |
| **Having received information about HCV** | | | | | | | | |
| Not informed (nether passively nor actively) | 1 (0-1) | 0.005 | 309 (79.6) | 79 (20.4) | <0.001 | 260 (67.0) | 128 (33.0) | 0.460 |
| Actively informed (± passively informed) | 0 (0-1) | | 72 (27.2) | 193 (72.8) | | 188 (70.9) | 77 (29.1) | |
| Only passively informed | 0 (0-1) | | 29 (39.2) | 45 (60.8) | | 48 (64.9) | 26 (35.1) | |

**Table S8. Risk factors, behaviors, and relationships with secondary outcomes**

| Characteristic | Attitude Score | | Having being tested for HCV | | | Sharing contaminated items | | |
|---|---|---|---|---|---|---|---|---|
| | **Median (IQR)** | **p** | **NO n=460 N (%)** | **YES n=353 N (%)** | **p** | **NO n=496 N (%)** | **YES n=231 N (%)** | **p** |
| **Sharing contaminated items** | | | | | | | | |
| No | 0 (0-1) | 0.770 | 280 (56.5) | 216 (43.5) | 0.965 | // | // | // |
| Yes | 0 (0-1) | | 130 (56.3) | 101 (43.7) | | // | // | |
| **Having ever had a sexual intercourse** | | | | | | | | |
| No | 1 (0-1) | 0.414 | 21 (60.0) | 14 (40.0) | 0.677 | 14 (53.8) | 12 (46.2) | 0.109 |
| Yes | 0 (0-1) | | 439 (56.4) | 339 (43.6) | | 482 (68.8) | 219 (31.2) | |
| **Frequency of condom use for occasional sex** | | | | | | | | |
| Never/almost never | 0 (0-1) | 0.200 | 85 (65.9) | 44 (34.1) | 0.131 | 74 (65.5) | 39 (34.5) | 0.849 |
| Sometimes | 1 (0-1) | | 37 (59.7) | 25 (40.3) | | 38 (67.9) | 18 (32.1) | |
| Often | 0.5 (0-1) | | 52 (53.1) | 46 (46.9) | | 62 (71.3) | 25 (28.7) | |
| Always | 0 (0-1) | | 71 (51.1) | 68 (48.9) | | 91 (71.1) | 37 (28.9) | |
| Do not have occasional sex | 0 (0-1) | | 215 (55.8) | 170 (44.2) | | 231 (67.3) | 112 (32.7) | |
| **Having a HCV positive partner** | | | | | | | | |
| No / Don't know | 0 (0-1) | 0.024 | 459 (58.3) | 328 (41.7) | <0.001 | 475 (67.7) | 227 (32.3) | 0.085 |
| Yes | 0 (0-0) | | 1 (3.8) | 25 (96.2) | | 21 (84.0) | 4 (16.0) | |
| **Having an addicted partner or housemate** | | | | | | | | |
| No / Don't know | 0 (0-1) | 0.230 | 451 (58.2) | 324 (41.8) | <0.001 | 472 (68.1) | 221 (31.9) | 0.762 |
| Yes | 0 (0-1) | | 9 (23.7) | 29 (76.3) | | 24 (70.6) | 10 (29.4) | |
| **Having HCV positive family members** | | | | | | | | |
| No | 0 (0-1) | 0.038 | 385 (57.5) | 285 (42.5) | <0.001 | 405 (67.3) | 197 (32.7) | 0.482 |
| Yes | 0 (0-1) | | 33 (38.8) | 52 (61.2) | | 54 (73.0) | 20 (27.0) | |
| Don't know | 1 (0-1) | | 42 (72.4) | 16 (27.6) | | 37 (72.5) | 14 (27.5) | |
| **Incarceration personal history** | | | | | | | | |
| No | 0 (0-1) | 0.052 | 460 (57.4) | 341 (42.6) | <0.001 | 488 (68.2) | 228 (31.8) | 0.747 |
| Yes | 0 (0-0) | | 0 (0.0) | 12 (100.0) | | 8 (72.7) | 3 (27.3) | |
| **Alcoholism personal history** | | | | | | | | |
| No | 0 (0-1) | 0.217 | 460 (56.9) | 348 (43.1) | 0.010 | 491 (68.0) | 231 (32.0) | 0.126 |
| Yes | 0 (0-0) | | 0 (0.0) | 5 (100.0) | | 5 (100.0) | 0 (0.0) | |
| **Being an Injective Drug User (IDU)** | | | | | | | | |

| | | | | | | | | |
|---|---|---|---|---|---|---|---|---|
| No | 0 (0-1) | 0.019 | 460 (58.0) | 333 (42.0) | <0.001 | 482 (68.1) | 226 (31.9) | 0.605 |
| Yes | 0 (0-0) | | 0 (0.0) | 19 (100.0) | | 14 (73.7) | 5 (26.3) | |
| **Having ever had an accidental wound caused by a syringe or other instrument contaminated by blood and/or other body fluids** | | | | | | | | |
| No | 0 (0-1) | 0.089 | 446 (97.0) | 14 (3.0) | <0.001 | 442 (69.0) | 199 (31.0) | 0.249 |
| Yes | 0 (0-1) | | 272 (77.1) | 81 (23.0) | | 54 (62.8) | 32 (37.2) | |
| **Being a blood donor** | | | | | | | | |
| No | 0 (0-1) | 0.053 | 324 (61.5) | 203 (38.5) | <0.001 | 328 (69.9) | 141 (30.1) | 0.182 |
| Yes | 0 (0-1) | | 135 (47.4) | 150 (52.6) | | 168 (65.1) | 90 (34.9) | |
| **Having received blood donation** | | | | | | | | |
| No | 0 (0-1) | 0.509 | 438 (57.9) | 319 (42.1) | 0.010 | 460 (67.9) | 217 (32.1) | 0.813 |
| At least one before 1992 | 0 (0-1) | | 4 (25.0) | 12 (75.0) | | 9 (69.2) | 4 (30.8) | |
| Only after 1992 | 1 (0-1) | | 18 (45.0) | 22 (55.0) | | 27 (73.0) | 10 (27.0) | |
| **Having Piercings** | | | | | | | | |
| No | 0 (0-1) | 0.304 | 114 (55.6) | 91 (44.4) | 0.094 | 140 (75.3) | 46 (24.7) | 0.108 |
| At least one performed independently | 0 (0-1) | | 23 (42.6) | 31 (57.4) | | 32 (65.3) | 17 (34.7) | |
| At least one performed professionally before 98 | 0 (0-1) | | 138 (55.6) | 110 (44.4) | | 151 (67.4) | 73 (32.6) | |
| At least one performed professionally after 98 | 0 (0-1) | | 184 (60.5) | 120 (39.5) | | 172 (64.7) | 94 (35.3) | |
| **Having Tattoos** | | | | | | | | |
| No | 0 (0-1) | 0.690 | 281 (56.1) | 220 (43.9) | 0.044 | 312 (70.0) | 134 (30.0) | 0.428 |
| At least one performed not professionally | 0 (0-1) | | 20 (40.0) | 30 (60.0) | | 28 (59.6) | 19 (40.4) | |
| At least one performed professionally before 1998 | 0 (0-1) | | 8 (53.3) | 7 (46.7) | | 10 (71.4) | 4 (28.6) | |
| At least one performed professionally after 1998 | 0 (0-1) | | 150 (61.5) | 94 (38.5) | | 144 (66.1) | 74 (33.9) | |
